# Trends in Adolescent Depression and Suicide Risk Screening and Symptom Monitoring in a Large Primary Care Network

**DOI:** 10.1016/j.acap.2025.103211

**Published:** 2025-12-20

**Authors:** Jason D. Jones, Chris Penney, Molly Davis, Ran Barzilay, Eamonn Tweedy, Fuchiang Tsui, Jami F. Young

**Affiliations:** Department of Child and Adolescent Psychiatry and Behavioral Sciences (JD Jones, M Davis, R Barzilay, and JF Young), Children’s Hospital of Philadelphia, Philadelphia, Pa; Department of Psychiatry (JD Jones, M Davis, R Barzilay, and JF Young), University of Pennsylvania Perelman School of Medicine, Philadelphia, Pa; PolicyLab (JD Jones, M Davis, and JF Young), Children’s Hospital of Philadelphia, Philadelphia, Pa; Department of Biomedical and Health Informatics (C Penney, E Tweedy, and F Tsui), Children’s Hospital of Philadelphia, Philadelphia, Pa; Department of Biostatistics (F Tsui), Epidemiology, and Informatics, University of Pennsylvania Perelman School of Medicine, Philadelphia, Pa; Department of Anesthesiology and Critical Care Medicine (F Tsui), University of Pennsylvania Perelman School of Medicine, Philadelphia, Pa

**Keywords:** adolescence, depression screening, primary care, suicide, symptom monitoring

## Abstract

**Objective:**

To examine long-term trends in adolescent depression and suicide risk screening and interim symptom monitoring in pediatric primary care (PC) following implementation of universal screening at well-visits.

**Methods:**

This retrospective cohort study examined electronic health record (EHR) data from 406,192 well-visits of 12 to 17-year-old pediatric patients from 2018 to 2024 in a large PC network in the United States. Screening was conducted using the Patient Health Questionnaire-9: Modified for Teens (PHQ-9-M). We evaluated trends over time in well-visit screening compliance, depression and suicide risk rates, and rates and timing of interim symptom monitoring between well-visits.

**Results:**

Screening compliance improved from 82.1% to 96.0% (χ^2^_MH_[1] = 15,996.93, *P* <.001; 89.2% 7-year compliance). Annual depression and suicide risk rates were generally stable over time, with highest rates observed in 2021. Less than 8% of patients screening positive for depression risk and less than 7% of patients screening positive for suicide risk received interim symptom monitoring in PC between well-visits. However, rates of interim screens increased over time ($\chi_{\text{MH}}^{2}\left[1\right]=240.74$, *P* <.001) and days from index well-visit to interim screen decreased by greater than 50%.

**Conclusions:**

In the years following implementation of universal depression and suicide risk screening in a large PC network, screening compliance increased significantly. However, low rates of interim symptom monitoring for patients screening positive indicate a gap in secondary prevention. Improving risk-based follow-up procedures, including interim screening, in PC represents a critical next step to enhance the preventive potential of universal screening.

Adolescent depression and suicide are major public health problems. Rates of adolescent depression, suicidal thoughts and attempts, and suicide deaths have increased in recent years, without commensurate increases in utilization of specialty mental health services.^1–4^ Pediatric primary care (PC)—the most common point of health care contact for youth—has become a critical setting for addressing mental health concerns.^5,6^ National guidelines recommend universal depression screening in PC at annual well-visits starting at age 12 years.^7–9^ Recommendations for universal suicide risk screening in PC are inconsistent: the American Academy of Pediatrics recommends universal screening starting at age 12, whereas the United States Preventive Services Task Force concluded there is insufficient evidence to recommend universal suicide risk screening.^10,11^ As a result, there is less data on universal suicide risk screening compared to depression screening. A study of pediatric medical inpatients found that screening for depression alone misses a substantial proportion of youth with suicide risk.^12^

As a population-based prevention strategy, universal screening aims to detect symptoms before they escalate, facilitate early intervention, and ultimately reduce long-term adverse outcomes. Despite national recommendations and the promise of universal screening, real-world implementation in PC remains inconsistent, with a wide range of screening rates reported in the literature.^13–15^ Most research on PC screening has been cross-sectional or focused on short-term changes in screening rates following quality improvement initiatives.^16^ There is limited research on longer-term trends in screening following implementation. Lewandowski et al.^15^ examined trends in depression screening in a large health maintenance organization and found significant increases in PC screening over 3 years.

Although screening in PC is a crucial first step in addressing youth mental health concerns, annual screenings may be insufficient for youth with depression and/or suicide risk. Ecological momentary assessment studies indicate substantial fluctuations in mood symptoms and suicidal ideation over time that are unlikely to be captured by annual screenings.^17,18^ Screening guidelines suggest ongoing monitoring of symptoms of patients identified as at-risk by initial screening.^7,8^ However, the optimal screening interval is not specified,^11^ and data are lacking on rates and timing of interim screens *between* annual well-visits to monitor symptoms.

In this study, we aimed to address these gaps by leveraging universal depression and suicide risk screening data from a large pediatric PC network that implemented universal screening procedures in late 2017. We analyzed 7 years of data (2018–2024) to evaluate trends over time in a) compliance with universal screening at well-visits, b) depression and suicide risk rates, and c) rates and timing of interim symptom monitoring in PC between well-visits.

## Methods

As part of Pennsylvania’s Quality Demonstration Grant for the Children’s Health Insurance Program Reauthorization Act, Children’s Hospital of Philadelphia (CHOP) began implementing electronic screens for developmental and behavioral issues from 2011 to 2014 using a phased implementation approach, including screening for depression at age 16 well-visits. At that time, PC practices received a mix of in-person trainings and electronically distributed materials, including psychoeducation about depression, information about screening and the measure used, descriptions of how to review and interpret the results, recommendations for speaking with families, treatment resources, and legal information. At the end of 2017, in response to national guidelines,^7–9^ CHOP transitioned to universal annual depression screening in PC starting at age 12.

For the present analyses, we extracted deidentified electronic health record (EHR) data for 12 to 17-year-old patients in the CHOP PC network, which includes 31 practices in urban and suburban areas of Pennsylvania and New Jersey. We analyzed data from January 1, 2018 to December 31, 2024, capturing 7 complete years following the implementation of universal screening. The CHOP institutional review board determined this study met criteria for exemption.

At CHOP, well-visit screening is conducted with the Patient Health Questionnaire-9: Modified for Teens (PHQ-9-M)^13,19^. In adults, a score of ≥10 is used as the clinical cutoff for depression on the PHQ-9.^19^ However, evidence suggests that a score of ≥11 is the optimal cutoff for adolescents.^20^ As such, at CHOP, depression scores are classified as borderline (5–10) or threshold risk (≥11). Suicide risk items assess past 2-week thoughts of death or hurting self, past month ideation, and lifetime suicide attempt. Patients were categorized as having suicide risk if they endorsed any of these items. The PHQ-9-M is completed electronically at well-visit check-in as part of standard clinical care. Results populate in the EHR for providers to review during the visit. If a patient endorsed borderline or threshold depression risk and/or suicide risk, the provider receives an EHR prompt to discuss the screening results with the patient. Interim screening prior to the next well-visit is done at the discretion of individual providers. At the time of well-visit screening, a provider may schedule a follow-up visit to reassess symptoms. Additionally, a provider could use a sick visit as an opportunity to rescreen.

### Statistical Analyses

#### Compliance with Annual Well-Visit Screening

For each year, from 2018 to 2024, screening rate was calculated as the number of well-visit encounters with a completed PHQ-9-M screening divided by the total number of well-visits. We also combined data across all years to calculate an overall 7-year screening rate. To evaluate changes in screening compliance over time, we used the Mantel-Haenszel (MH) chi-squared test, which tests if there is a linear association between 2 categorical variables (study year and screening status). The analysis also provides odds ratios (ORs) reflecting the odds of being screened in each year relative to the reference year (2018).

#### Interim Symptom Monitoring Between Well-Visits

For each year from 2018 to 2023, we identified patients who had a PHQ-9-M well-visit screen (index well-visit) and then had a subsequent well-visit during the study timeframe (2018–2024). We limited index well-visits to through the end of 2023 to allow time for patients to have a subsequent well-visit in 2024. Among these patients with 2 well-visits, we analyzed rates and timing of interim screening that occurred in PC between these well-visits. We limited the sample to patients with 2 well-visits to capture patients who had the opportunity for interim symptom monitoring. Otherwise, follow-up rates could be skewed by patients who may have aged out of pediatric PC or who did not return for a well-visit in this network. Among patients who had an interim screen between well-visits, we calculated the average days from index well-visit to interim screen. Changes in rates of interim symptom monitoring over time were evaluated using the Mantel-Haenszel chi-squared test. Changes in timing of interim symptom monitoring were evaluated by linear regression using year as a categorical predictor and days to interim screening as the outcome. We examined changes in interim screening rates and timing among all patients as well as specifically among patients who screened positive for depression or suicide risk, as we would expect higher rates of follow-up among patients who screen positive.

## Results

Data included 406,192 well-visits (161,030 unique patients) from 2018 to 2024. Patients were 14.79 years (SD = 1.73), on average; 49.4% female; 52.2% White, 27.2% Black, 4.8% Asian, 15.9% other races; 8.3% Hispanic/Latino; 25.4% were Medicaid-insured.

### Well-Visit Screening

From 2018 to 2024, the PHQ-9-M was administered at 362,318 well-visits (89.2% 7-year screening compliance). From 2018 (82.1%) to 2024 (96.0%), compliance with universal screening increased significantly, $\chi_{\text{MH}}^{2}\left[1\right]=15,996.93$, *P* < .001. Examination of yearly screening rates and ORs indicates that significant increases in screening compliance began in 2021. Screening rates by year, ORs reflecting the odds of being screened each year relative to 2018, and depression and suicide risk rates are reported in Table 1.

### Interim Symptom Monitoring *Between* Well-Visits

Interim screening rates before next well-visit were low across the whole study time frame, but increased significantly over time for all patients ($\chi_{\text{MH}}^{2}\left[1\right]=240.74$, *P* < .001), patients with borderline depression symptoms ($\chi_{\text{MH}}^{2}\left[1\right]=231.28$, *P* < .001), patients with threshold depression symptoms ($\chi_{\text{MH}}^{2}\left[1\right]=135.56$, *P* < .001), and patients with suicide risk ($\chi_{\text{MH}}^{2}\left[1\right]=108.78$, *P* < .001). Examination of yearly interim screening rates and ORs indicated that significant increases in interim screening began in 2020. Interim screening rates by year and ORs relative to 2018 are reported in Table 2. Average days to interim screen decreased dramatically over time (56%–62% reduction). Regression analyses revealed that year was a significant predictor of days to interim screen. Coefficients indicated that days to interim screen began to significantly decrease in 2020/2021 (see Table 3).

## Discussion

Results offer critical insights into the long-term implementation of universal depression and suicide risk screening as a population-based preventive strategy within pediatric PC. Over 7 years, screening rates rose to > 95%, reflecting strong adoption and sustainment of universal screening when supported by standardized workflows, EHR integration, and system-level prioritization. These findings extend previous research on depression screening in PC^15,16^ and provide longitudinal evidence of real-world implementation in a large PC network.

The rise in screening rates was likely driven by several converging factors, including both internal changes within the health system and broader shifts in the public health landscape. Internally, the CHOP PC network—spanning more than 30 practices across 2 states—required time to fully roll out universal screening procedures, build provider awareness, and optimize informatics and clinical workflows. Large systems do not achieve uniform implementation immediately; maturation of processes and normalization of new workflows naturally take time. Externally, national attention to the youth mental health crisis^21^ intensified during and after the COVID-19 pandemic, which likely increased clinician vigilance regarding adolescent mental health concerns. Updates to national guidelines, policy changes, and the inclusion of depression screening as a health care quality metric also strengthened the expectation for routine screening.

While high annual screening compliance demonstrates the feasibility and sustainability of integrating depression and suicide risk screening into routine care, the persistently low rates of interim symptom monitoring between well-visits highlight a key gap in care. Although rates improved modestly over time for patients who screened positive for depression or suicide risk, interim symptom monitoring in PC is still far from routine. However, days from index well-visit to interim screening decreased dramatically over time, likely reflecting maturation of workflows and increased clinician familiarity with managing adolescent depression and suicide risk in PC. Faster reassessment improves continuity of care, increasing the likelihood of capturing symptom escalations, and provides the opportunity for earlier intervention.

These findings underscore the need for prevention-focused innovations in PC that go beyond initial detection to include systematic, risk-informed monitoring which can support timely intervention. Provider education about the utility of the PHQ-9-M as an ongoing symptom monitoring tool could be beneficial. Automating administration of the PHQ-9-M at all visits, not just well-visits, for patients who previously screened positive would increase interim screening rates without adding significant burden. Strengthening these components of the care continuum is essential to realizing the full preventive potential of universal screening and mitigating the downstream consequences of adolescent depression and suicidality.

This study has limitations. Data were derived from a single health system, which may limit generalizability to other settings. Follow-up was defined narrowly as interim PHQ-9-M administration; other forms of follow-up—such as PC visits without PHQ-9-M screening or referral to behavioral health services—were not captured, likely underestimating follow-up care received. The COVID-19 pandemic influenced both mental health disorder prevalence and clinical operations, which likely impacted observed changes over time. We capitalized on retrospective EHR data from over 400,000 well-visits to obtain a broad picture of screening trends over time. However, EHR data inherently limit the ability to capture fine-grained contextual information, such as specific reasons why screening did or did not occur at individual encounters. Similarly, we could not assess nuances in how providers interpreted screening results or made decisions regarding symptom monitoring. Additionally, we limited our interim screening analyses to patients with at least 2 well-visits. As a result, patients who were screened once and did not return for a well-visit were not captured in these analyses.

Universal screening has clear value as a population-based prevention strategy: it identifies large numbers of adolescents with depressive symptoms and suicide risk at an accessible point of care. Yet, annual screening alone is insufficient. Without timely follow-up, systems fail to capitalize on prevention and early intervention opportunities. Improving post-screening care pathways—through structured follow-up protocols, team-based care, and integration of behavioral health—represents the next frontier in closing the gap between detection and management of youth mental health concerns in PC.^14^

## Figures and Tables

**Table 1.** Screening and Risk Rates at Annual Well-Visits

Year	Well-Visits (N)	Screening Rate*	ORs^†^	PHQ-9-M Borderline (5–10)	PHQ-9-M Threshold (≥11)	PHQ-9-M Suicide Risk^‡^	Item 9	Item 12	Item 13
2018	50,749	82.1%	–	22.9%	5.6%	6.8%	4.4%	2.3%	2.9%
2019	55,282	81.6%	0.97	22.2%	5.5%	6.6%	4.0%	2.1%	3.2%
2020	51,007	80.0%	0.87	23.7%	6.4%	7.2%	4.5%	2.3%	3.4%
2021	59,495	89.2%	1.81	25.4%	7.5%	8.3%	5.2%	2.9%	3.8%
2022	58,802	94.9%	4.03	23.4%	6.2%	7.8%	4.3%	2.8%	4.1%
2023	63,250	96.4%	5.88	22.5%	5.5%	7.3%	3.9%	2.4%	3.8%
2024	67,607	96.0%	5.23	21.9%	4.9%	6.8%	3.5%	2.3%	3.6%

* Screening rate refers to the percentage of eligible patients who were screened at a well-visit (screened patients/total number of well-visits).

† ORs = odds ratios reflecting odds of being screened each year relative to 2018.

‡ Suicide risk on the PHQ-9-M is operationalized as a response > 0 on item 9 (“Thoughts that you would be better off dead, or of hurting yourself in some way?”) and/or an affirmative response to either item 12 (“Has there been a time in the past month when you have had serious thoughts about ending your life?”) or item 13 (“Have you ever, in your whole life, tried to kill yourself or made a suicide attempt?”).

**Table 2.** Interim Symptom Monitoring Between Well-Visits

Year	Overall		PHQ-9-M Borderline (5–10)		PHQ-9-M Threshold (≥11)		PHQ-9-M Suicide Risk	
	Rate* %	ORs^†^	Rate %	ORs	Rate %	ORs	Rate %	ORs
2018	1.2	–	1.6	–	1.7	–	1.9	–
2019	1.0	0.83	1.3	0.78	1.3	0.76	1.5	0.82
2020	1.7	1.48	2.9	1.78	3.8	2.18	2.9	1.50
2021	2.6	2.13	4.5	2.73	6.2	3.56	5.4	2.85
2022	2.6	2.20	5.1	3.24	7.4	4.46	6.4	3.48
2023^‡^	2.0	1.70	4.3	2.74	7.4	4.60	5.8	3.24

* Interim symptom monitoring rate refers to the percentage of patients who were screened at a well-visit and then received another screening before their next well-visit.

† ORs = odds ratios reflecting odds of receiving an interim screen before the next well-visit for each year relative to 2018.

‡ It is possible that patients with an index well-visit in 2023 had another PC visit after December 31, 2024, which goes beyond the available data. As a result, figures reported for 2023 may slightly underestimate the true interim screening rate. We included 2023 to utilize all available data.

**Table 3.** Average Days to Interim Symptom Monitoring Over Time

Year	Overall			PHQ-9-M Borderline (5–10)			PHQ-9-M Threshold (>11)			PHQ-9-M Suicide Risk		
	Days^a^ *M* (*SD*)	β^b^	95% CI	Days *M* (*SD*)	β	95% CI	Days *M* (*SD*)	β	95% CI	Days *M* (*SD*)	β	95% CI
2018	443 (236)	–	–	423 (233)	–	–	386 (251)	–	–	392 (150)	–	–
2019	470 (252)	27	0.65, 53	418 (252)	−5	−55, 44	309 (185)	−77	−171, 17	416 (280)	24	−55, 102
2020	379 (197)	−64*	−87, −40	358 (215)	−64*	−105, −23	310 (191)	−76	−149, −3	326 (187)	−66	−134, 1
2021	326 (176)	−117*	−138, −96	296 (188)	−127*	−164, −90	270 (194)	−116*	−183, −49	282 (190)	−110*	−169, −51
2022	257 (151)	−186*	−208, −165	221 (156)	−202*	−239, −165	149 (112)	−205*	−272, −138	191 (142)	−201*	−259, −143
2023^b^	196 (114)	−247*	−269, −225	175 (111)	−248*	−286, −210		−238*	−306, −169	150 (116)	−242*	−302, −182
		*F* = 193.64, *P* < .001			*F* = 67.81, *P* < .001			*F* = 20.41, *P* < .001			*F* = 30.39, *P* < .001	

95% CI indicates confidence intervals for regression coefficients.

* *P* < .01

a Days represents average days from index well-child visit to interim screen before next well-visit

b Coefficients represent change in average days to follow-up screening relative to 2018

## What’s New

Longitudinal analysis of trends in adolescent depression and suicide risk screening and symptom monitoring in large primary care network. Screening compliance was high (89.2%). Most patients who screen positive do not receive follow-up monitoring, highlighting a gap in care.
